# Service Leadership through Serving Minority Adolescents in Rural China Using a Rural Version of a Positive Youth Development Program

**DOI:** 10.1007/s11482-022-10098-0

**Published:** 2022-09-16

**Authors:** Zheng Zhou, Liping Mu, Shaojie Qi, Daniel T.L. Shek

**Affiliations:** 1 Research Institute of Social development, Southwestern University of Finance and Economics, Sichuan, China; 2 Department of Applied Social Sciences, The Hong Kong Polytechnic University, Hung Hom, Hong Kong

**Keywords:** Service-learning, Positive youth developmennt, Minority adolescents

## Abstract

With the emerging service economy globally, nurturing university students to be service leaders possessing the leadership qualities of competence, character and care is of great importance. Through service-learning (SL), the academic learning of the students on Service Leadership can be consolidated. In the present study, we piloted a SL subject on Service Leadership in a university in the Southwestern part of China. After learning the basic academic knowledge on Service Leadership, seven students provided service for the Yi minority adolescents in Liangshan using the rural version of the program. To understand the impact of SL on the service recipients, a single group pretest-posttest design was used to assess the changes in Yi minority adolescents (N = 79) before and after they received the service. We also conducted subjective outcome evaluation of the service providers (the university students providing the service) and service recipients (Yi adolescents). Results showed that the service recipients had significant positive changes in the outcome indicators and they had positive perceptions of the program. The qualitative evaluation findings are also encouraging. The findings provide evidence supporting the use of SL in nurturing service leadership qualities in university students in mainland China.

## Introduction

A mission of universities is to educate university students and nurture them to be better citizens. In fact, most university graduates become important leaders in various fields in the society. Under the manufacturing economy in the past centuries, we expect universities to train graduates who can manage and lead in the assembly lines. However, with the global shift from manufacturing to service production, we need leadership that suits the need of service economies. According to Shek et al. (2015), the expectations and requirements of leadership are substantially different in the manufacturing and service economies. In particular, Leadership qualities of generic competencies, moral character and caring dispositions are intrinsic to effective service leaders (Shek & Lin, 2015, Shek et al., 2018; Zhu & Shek, 2021). While there is an overlap in leadership competencies under manufacturing and service economies (e.g., problem-solving skills and resilience), some desired competencies are different. For example, interpersonal communication skills are particularly important in service economies because simply “giving orders” and “taking orders” are not adequate. Besides, moral character (e.g., integrity and fairness) and care play a very important role in service economies (Shek & Lin, 2015). In short, service leadership is defined as the leadership attributes cherished under the service economy, particularly generic competencies, character, and care (Shek et al., 2018; Shek, Chung & Zhu 2020).

How can universities nurture service leadership qualities in university students? Primarily, we can design classroom-based service leadership subjects in the context of General Education to serve this purpose. While classroom-based teaching and learning could be used (Zhu & Shek, 2021), one limitation is that there is no opportunity for students practice and consider their service leadership and reflect on the question of whether they are service leaders. To overcome this challenge, we may use service-learningas a tool to consolidate the academic learning of the students. Service-learning is a pedagogy that incorporates community service in the academic learning of the students (Ehrlich, 1996). According to Bringle et al., (2006), service-learning is an educational experience where students design and organize community service based on the subject knowledge to address community needs. Ehrlich (1996) conceptualized service-learning (SL) as pedagogies which “link community service and academic study so that each strengthens the other. The basic theory of service-learning is Dewey’s: the interaction of knowledge and skills with experience is key to learning” (*pp*. xi).

The Hong Kong Polytechnic University has adopted both approaches to cultivate undergraduate students’ service leadership qualities. First, we have developed a classroom-based subject entitled “Service Leadership” to introduce the subject matter of service leadership which includes leadership competencies (such as adversity quotient, emotional quotient and spiritual quotient), character and care. In the subject, we use experiential teaching and learning approaches to maximize teacher-student and student-student interactions. We also encourage students to reflect on their service leadership qualities and apply their learning to real life. Numerous evaluation studies have shown that the subject is able to promote the well-being and purpose of the students (Leung et al., 2021; Li & Shek, 2019; Lin & Shek 2019; Shek et al., 2022; Zhu et al., 2021). This subject has received the Bronze Award (Ethical Leadership) in 2016 and Gold Award (Nurturing Student Well-Being and Purpose) in 2021 under the QS Reimagine Education Awards. It was also awarded the UGC Teaching Award in 2018.

We have also developed another service-learning subject entitled “Service Leadership through Serving Children and Families with Special Needs”. In this subject, students first learn the basic theories and concepts on service leadership. Then they apply the knowledge gained in service-learning project through which they reflect on their leadership competencies, character and care. Past research has shown that SL can bring positive influence on students’ service leadership qualities, positive youth development (PYD) attributes and well-being under conditions with and without COVID-19 (Celio et al., 2011; Lin & Shek, 2021; Yorio & Ye, 2012). This subject has also attracted many prestigious teaching awards (e.g., Gold Award (Sustainability) under QS Reimagine Education Awards in 2017.

With specific reference to mainland China, we notice that there are very few leadership subjects in the university context, with most of the existing leadership courses offered by colleagues in the Faculty of Business. Besides, there is no known leadership subjects focusing on service leadership. Furthermore, service-learning in higher education is comparatively new in mainland China. Though the concept of “holistic youth development” has been proposed for years by the Chinese government, Chinese education system has long been criticized as overemphasizing academic outcomes rather than cultivating practical competence in students (Chen & Zhang, 2021). For instance, the educational pedagogy in Chinese higher education overwhelmingly focuses on classroom teaching and the evaluation of students is mostly based on the course grade. The lack of social practice and community service in the learning environment leads to the detachment of students to the community, as well as the low social responsibility among the undergraduates (Chen & Zhang, 2021). For the Master of Social Work programs in China, researchers also pointed out a number of deficiencies in the developmental outcomes of the students: lack of integration of theories and practice, insensitivity of the students to social needs, and low professional identity (Chen & Zhang, 2021; Li et al., 2011; Yao & Jiang, 2021). In particular, Yao & Guo (2015) pointed out that the education system in mainland China has not attached enough importance to SL so that the design of service-learning subjects is mostly not professional and students usually are not able to fully devote and reflect during the service activities. For instance, a recent evaluation of a service-learning subject at a university in China found the undergraduates did not show significant improvements in their social competence and self-evaluation, though their research competence was improved (Zhu, 2020). In short, although some universities in mainland China offer service-learning subjects, the effort is unsystematic and evaluation is not commonly conducted. Against this background, it is very necessary and meaningful to introduce the pedagogy of service-learning to Chinese higher education and conduct evaluation of the related initiatives (Hong et al., 2020; Peng et al., 2009).

In the present study, we offered a service-learning subject at the Southwestern University of Finance and Economics, modeling after the “Service Leadership through Serving Children and Families with Special Needs” which has been shown to be effective in promoting the developmental outcomes of the service providers (i.e., university students) and service recipients (Lin & Shek, 2019; Shek et al., 2020). By taking the course, students are expected to: (a) acquire knowledge of service leadership, (b) link their knowledge of adolescent social work with service experiences, (c) deal with the recipients by providing field services, and (d) reflect on their service leadership qualities after the service activities.

This service-learning subject includes 9-hour online lectures on service-leadership, 15-hour seminars for service planning, preparation and group presentation, 80-hour service implementation, 10-hour post-field work study and reflection. The students were required to take a related course on child and adolescent social work before registering for the course. The online lectures aim to help students understand the core qualities of service leaders in terms of competence (e.g. intrapersonal and interpersonal competencies), moral character (e.g., moral character, ethical and moral issues when serving others), and caring disposition. After learning the basic concepts of service leadership, students were divided into small groups to develop the service plan for minority youth (Yi adolescents) based on the rural version of the Tin Ka Ping P.A.T.H.S. Prorgam in China. After the service, group sessions were conducted to help students reflect on their service leadership qualities and to evaluate the effectiveness of experiential learning activities.

## Minority Youth in Mainland China and the Relevance of Positive Youth Development (PYD) Programs

As of 2015, there were 31.11 million ethnic minority children aged 0–17 in China, accounting for 26.5% of the total ethnic minority population (UNICEF, 2018). Among them, the number of Yi youths is 1.57 million, accounting for 8.91% of the total population of minority youth. In China, the Yi population is mainly distributed in Liangshan Yi Autonomous Prefecture, which is one of the extremely poor areas due to poor natural conditions such as the lack of farmland. Teenagers in poverty are at-risk in cognitive ability, psychosocial competence, and academic achievement (Dashiff et al., 2009; Yang, 2018). Research studies suggest that Yi teenagers are exposed to a number of developmental challenges, such as poor mental health (Ma & Guo, 2017), poor academic competence (Liu, 2014), low self-evaluation (Chen et al., 2007), negative emotional experience (Zhou & Gong, 2011), and high rate of dropout and school weariness (Deng, 2019). In recent years, the Chinese Government has attached great importance to improving the living status of Yi people in Liangshan and constantly pushed targeted poverty alleviation at a deeper level. Relocation has become an important strategy for poverty alleviation and human development in the Yi ethnic area. However, the problems of community integration and community adaptation caused by the relocation have become new issues that plague the community residents and teenagers (Wu & Liu, 2020).

How can we foster the holistic development of Yi adolescents? With reference to the developmental problems of Yi adolescents, we definitely need prevention programs. However, designing a prevention program for one specific developmental problem is not efficient and there is a need to understand the origin of adolescent developmental problems. In fact, it is argued that many adolescent developmental problems emerge with a common origin of lacking developmental assets such as psychosocial competencies. In the past few decades, a growing body of research has focused on fostering positive development of youth (Leman et al., 2017; Qi et al., 2020; Smith et al., 2013; Synder & Flay 2012). Youngsters can achieve positive developmental outcomes through building up external assets (such as family bonding and community support) and/or internal assets (such as skills and values), (Benson et al., 1998; Shek et al., 2019, 2020; Catalano et al., 2004) proposed a positive youth development model consisting of 15 constructs, such as having good bondage with healthy individuals, life meaning, and psychosocial competencies. Chinese studies also showed that PYD attributes improve adolescent life satisfaction, and are negatively related to depression and hopelessness (Zhou et al., 2020, 2021; Zhu & Shek, 2020a, b).

In reality, PYD intervention programs have been implemented in different countries and regions (Taylor et al., 2017) with most of them implemented in Western contexts (Wiium & Dimitrova, 2019). Recently, PYD programs implemented in Hong Kong and mainland China also achieved success in improving adolescent holistic development (Shek et al., 2019; Shek & Wu 2016; Zhu & Shek, 2020a). For example, the P.A.T.H.S. Prorgam has been implemented in more than 200 high schools and communities in Hong Kong since 2005 (Shek & Wu, 2016). The follow-up evaluations of the project using a quasi-experimental design showed that the program promoted experimental school students’ psychological competence and reduced delinquency in the experimental school compared with control school students (Ma et al., 2018, 2019; Shek & Ma, 2012).

Based on the P.A.T.H.S. Project, an adapted PYD program was introduced to eastern China on a large scale since the 2011/2012 academic year (Zhu & Shek, 2020b). With the financial support of Tin Ka Ping Foundation,, we have developed the Tin Ka Ping P.A.T.H.S. Program to be used in high schools in mainland China (Shek, Zhu, Leung et al., 2019). Objective oucome evaluation findings showed that the program had a positive effect on the participants’ development (Shek et al., 2014; Zhu & Shek, 2020a, b). Besides, subjective outcome evaluation (Shek & Law, 2014) and qualitative evaluation (Shek, Zhu, Leung, et al., 2019) also showed that the program was beneficial to the development of the program participants.

Despite the positive outcomes of the Project P.A.T.H.S. and Tin Ka Ping P.A.T.H.S. Project in mainland China, it is important to examine how well the programs are implemented in different contexts such as the rural context (Ma et al., 2019). First, as most PYD programs in Hong Kong and mainland China are applied primarily in the school context, whether the program is applicable in a community-based context is worthy to determine. Second, it is valuable to determine how the positive effects of PYD programs vary by the recipients from different socio-economic and ethnic backgrounds, because minority youth face more developmental challenges due to their social status, such as low family income and poor educational resources (Motti-Stefanidi, 2017). However, limited empirical studies have investigated the positive youth development for minority youth (Lerner, 2017).

As the curriculum manuals in the Tin Ka Ping P.A.T.H.S. Project is mainly used in the urban or town contexts, the materials may not be fully appropriate for the rural area of China. Hence, we formed a collaborative research team involving colleagues of the Hong Kong Polytechnic University University and Southwestern University of Finance and Economics to develop the rural version of the Tin Ka Ping Rural program (TKP-Rural P.A.T.H.S). In this service-learning project, with the supervision of researchers from the Hong Kong Polytechnic University and SWUFE, students who registered for the service-learning course make necessary modifications of the Tin Ka Ping P.A.T.H.S. Program (TKP P.A.T.H.S) to fit the cultural and socio-economic background of Yi communities. Major adaptations include the wording and course materials: (1) wording used in urban context were modified to rural context; (2) teaching materials with urban socio-cultural features were replaced by materials which are suitable for the rural context (such as the role model from urban background introduced to the students were replaced by another successful role model from rural area).

Through the service-learning project, we expected the service providers (i.e., university students) to consolidate their learning (i.e., the importance of leadership competencies, character and care in providing the service) and have reflection on their own Service Leadership qualities. Conceptually speaking, the content of TKP Rural …Project well aligned with the Service Leadership Theory. For example, psychosocial competencies covered in Project are well aligned with the “competence” element in Service Leadership Theory. “Caring for others” in the Project…e.g., social competence, spirituality and prosocial norms) also resonates with the element of “Care” in Service Leadership Theory. The focus on “character” in service leadership also aligns with “moral competence” and “spirituality” in positive youth development literature.

## The Present Study

This study explored the impact of a service-learning project arising from a Service Leadership subject implemented in a Yi community in southwestern China. This service-learning project was conducted in two relocation and poverty alleviation Yi communities in Meigu County which is located in the hinterland of Daliang Mountain. This area has steep slopes and deep valleys, as well as lagging road traffic and public services, making it once the most impoverished and difficult poverty alleviation county in Liangshan Prefecture. Until May 2020, all the county’s relocation projects for poverty alleviation had been completed, and 53,223 people from 10,694 poor households have successfully left the mountain area. As such, how to ensure that these relocated community residents, particularly young people, adapt to the new lifestyle and participate in community construction has become a priority issue to be examined. Therefore, conducting PYD programs in the community for Yi adolescents would be helpful to their adjustment at this critical time.

The present study attempted to address the following research gaps in the literature. First, though service-learning courses have been implemented in universities in mainland China, most of the service-learning subjects were provided in urban communities. There are no service-learning projects providing service for Yi adolescents in the mountain area in China. As Yi adolescents from one of the poorest area in China is facing different kinds of developmental challenges, it is valuable to implement service-learning project to serve these adolescents in need.

Second, there is a lack of systematic evaluation of service-learning projects in mainland China. Primarily, few service-learning subjects in mainland China adopted different evaluations to triangulate the research findings. In the present study, we used systematic evaluation to assess the effectiveness of the service-learning subject, including objective and subjective evaluation, as well as quantitative and qualitative evaluations. Moreover, the importance of evaluation based on the service recipients is largely ignored (Shek et al., 2020). As subjective and objective outcome based on the service recipients is an important way to evaluate the effectiveness of a service-learning subject, the present study evaluated the service-learning project not only from the service providers but also from the service recipients. The objective outcome evaluation of the present study would adopt a one-group pretest-posttest design to test the changes of the adolescents in their positive youth development attributes. The one-group pretest-posttest design has been commonly used higher education sector to identity the change of students who participated in a program (Felix, 2016; Gregorová et al., 2016; Shek & Sun, 2012; Shek et al., 2022). For example, Felix (2016) investigated whether the students enrolled in online foreign language courses improved their course competencies. Shek et al., (2022) and Lin, Shek and Li (forthcoming) also evaluated the effect of a service-learning project on the service providers based on the change of the students’ pretest and posttest scores of positive youth development and service leadership qualities. In addition, the relationship between objective outcome and subjective outcome evaluation based on the service recipients is understudied. The present study addressed this research gap by investigating how the objective evaluation was associated with the subjective evaluation of the service recipients.

Third, there is no study on service-learning subjects using a positive youth development approach in the rural and ethnic areas of China for Yi adolescents. First, though the service-learning subject we adopted achieved success in Hong Kong, how well such modified service-learning subjects for serving the need of Yi adolescents are implemented in the rural ethnic area of China is still unknown. Second, despite the positive outcomes of the Project P.A.T.H.S. in Hong Kong and Tin Ka Ping P.A.T.H.S. Project in mainland China, it is important to examine how the rural version of Tin Ka Ping P.A.T.H.S. project benefits the rural Yi adolescents. As minority youth face even more developmental challenges, it is valuable to examine how the positive effects of service-learning subject vary by the recipients from different socio-economic and ethnic backgrounds.

We asked several research questions in this study:

1. Are there are any changes in the program participants after participating in the program? Based on the previous studies (Shek et al., 2021; Shek & Wu 2016; Zhu & Shek, 2020a), we expected that Yi adolescents would change in the positive direction after joining the program (Hypothesis 1).
2. What are the holistic perceptions of Yi adolescents of the service-learning Project? Based on the previous findings that the service recipients are positive about the program as a whole (Shek, Yang, et al., 2021), we expected that a majority of the participants would have positive views of the program as a whole (Hypothesis 2).
3. What are the perception of the program participants of the sessions in the intervention program? According to previous studies, students viewed the lessons positively (Lin & Shek, 2021). We expected that a majority of the responses given by the students would be positive (Hypothesis 3).
4. Is objective outcome evaluation related to subjective outcome evaluation? According to previous study (Shek, 2014), we hypothesized that the change in objective outcome evaluation would predict subjective perceptions of the program as a whole (Hypothesis 4).
5. What are the views of the service providers (i.e., university students) on the program, their own performance and benefits? Based on previous findings (Shek et al., 2020), we expected that the service providers would show positive perceptions of the program, their own performance and benefits (Hypothesis 5).

## Methods

### Participants and Procedures

In the academic year of 2020/2021, a service learning subject entitled as “Service Leadership through Serving Children and Families with Special Needs” was offered to graduate students at the Southwestern University of Finance and Economics. This subject was mirrored after a similar subject offered at the Hong Kong Polytechnic University. The content of the subject included: (1) 9-hour online lectures on service leadership including the concepts and theories of service leadership, and positive youth development models; (2) 15-hour seminars of collaborative learning for service planning and preparation; and (3) 9-hour service practice for adolescents at a near middle school under the guidance of the lecturer. The postgraduate students who registered the subject designed and used a positive youth development program based on the rural version of the Tin Ka Ping P.A.T.H.S. project to service Yi adolescents from an extremely poor area in Sichuan Province at southwestern China. The Yi adolescents receiving the service were recruited from two Yi minority communities at the beginning of July 2021. Researchers explained the purpose and procedures of the project to the adolescents and consent forms were obtained from both the adolescents and their parents. Power analysis using G*power 3.0 showed that the improvement of adolescents’ outcome measures before and after the service could be detected with 80% power if the sample size reaches 71 (one tail, α = 0.05, assuming the effect size would be 0.3). Considering some participants might withdraw from the project, we finally recruited 92 Yi adolescents to join the course.

A 7-day positive youth development service (2 h per day) (Table 1) was provided by 7 students in Master of Social Work to the Yi adolescents in July 2021. We adopted a one group pretest/posttest design to access the objective change of the Yi adolescents which was commonly used in previous higher education research including service-learning (Felix, 2016; Gregorová et al., 2016; Shek & Sun, 2012; Shek et al., 2022). Adolescents were asked to complete the Chinese Positive Youth Development Scale before and after the 7-day course. Besides, because using various evaluation methods as triangulation has been commonly used in positive youth development programs (van de Vijver & He 2017; Shek et al., 2020) to increase the credibility and validity of research findings, the present study used qualitative evaluation and subjective evaluation to triangulate the effectiveness of the subject on the recipients as previous study. After each section, adolescents were asked to write down their perception of the effectiveness of the course as a qualitative evaluation of each section (e.g.“What did I learn in the course?”). At the end of the project, the subjective outcome evaluation questionnaire of the whole course was also distributed to the program participants. Finally, 79 Yi adolescents participated in all sessions and completed the pretest, posttest and subjective outcome evaluation (*M*_age_ = 14.31, *SD*_age_ = 1.29, 48 females). The service providers (*N* = 7) also responded to the subjective evaluation form when the service ended.

**Table 1:** A 7-day positive youth development service-learning project

	Units	Constructs	Objectives	Courses reflection questions
1	Introduction	Initial structuring	To introduce the goals, rationales, and general format of the courses. To establish the connection between students, teachers and form an agreement.	
2	EC1.1 EC1.2	Emotional Competence	To understand the meaning of emotion. To try to show different emotions with abundanrt words and enhance the emotional management ability.	What should I do after having a bad mood?
3	BO1.1 RE1.1	Bonding Resilience	To establish positive connections between students and mentors, helpful friends, and family members	What did I learn in the course?
4	SE1.1 SE1.2	Self-efficacy	To identify and assess self-efficacy from various aspects, such as study, clothing and living habits.	What are the implications for achieving success?
5	ID1.1 BF1.1	Clear and Positive Identity Beliefs in the Future	To encourage students to find their life direction with a positive and optimistic attitude. To emphasize the importance of future beliefs.	How did the course help me?
6	PN1.2 PI1.1	Prosocial Norms Prosocial Involvement	To guide students to understand their community and culture, and find a sense of belonging and identity to their hometown and community. To facilitate students to understand what they can contribute to their community and the positive effects of it.	What can I do for the development of my hometown?
7	End course	Summary	To summarize students ‘growth experience and affirm the change of students’ growth.	

### Evaluation Based on the Service Recipients

#### Objective Outcome Evaluation:

Before and after the service, participants completed a set of measures including positive youth development qualities and life satisfaction.

For PYD qualities, adolescents were asked to complete a short version of Chinese Positive Youth Development Scale (Shek, Siu, et al., 2007) (pretest Cronbach’s α = 0.95; posttest Cronbach’s α = 0.96). The CYPDS had 43 items including bonding (4 items, e.g. “I believe my family will help me whenever I need”), resilience (3 items, e.g. “I believe problems in life can be solved”), social competence (3 items, e.g. “I know how to communicate with others”), recognition for positive behavior (3 items, e.g. “My classmates will accept and support me when I help others”), emotional competence (3 items, e.g. “When I am unhappy, I can appropriately express my emotions”), cognitive competence (3 items, e.g. “I know how to find the causes of and solutions to a problem”), behavioral competence (3 items, “I can express views that are different from others”), moral competence (3 items, “I fulfill my promises”), self-determination (3 items, “I am confident about my decisions”), self-efficacy (2 items, e.g. “I can manage to do the things that I decide to do”), clear and positive identity (3 items, e.g.“I am confident”), beliefs in the future (3 items, e.g. “I have the confidence to solve any difficulty in the future”), prosocial involvement (3 items, e.g. “I will try my best to contribute to the school and the society”), prosocial norms (3 items, e.g. “I conform the rules of the school”), and spirituality (3 items, “I have found my purpose in life”). Participants rated their answer from 1 (strongly disagree) (1) to 6 (strongly agree) except the 3 items of spirituality. For the 3 items measuring spirituality, participants rated the items from 1 to 7.

For life satisfaction, it was measured by 5 items of the “Satisfaction with Life Scale” (SWLS) (Diener et al., 1985). Participants were asked to rate the items in the scale from 1 (strongly disagree) to 6 (strongly agree) to report their general satisfaction with their life, as the cognitive aspect of subjective well-being. The sample item was “the conditions of my life are excellent”. The internal consistency of this scale is 0.70 (pre-test) and 0.73 (post-test).

#### Subjective Outcome Evaluation

After the service, we used the Subjective Outcome Evaluation Scale-Service Recipients (SOES-SR) (Shek, Yang et al., 2021) to assess the perception of the service recipients of the course content, instructors, and the perceived benefits as a whole. The perception of the course content included 10 items such as “The design of the course content is good” “In general, I like this course” etc. The perception of the instructors included “The instructor is well-prepared” “I have a positive evaluation of the instructor’s performance” etc. The two measures were a 6-point scale and the answers were ratedfrom “strongly disagree” (1) to “strongly agree” (6). The perception of the benefits in the course included 16 items that assess the subjective improvement in positive youth development qualities, e.g. “The course improves my capacity to get along with others” “The course reinforces my confidence”. This measure was a 6-point scale and students were asked to rate their opinion from “not helpful at all” (1) to “very much helpful” (5). Three additional questions were added to the questionnaire, including: (1) Will you recommend this course to your friends? (2) Will you participate in this course in the future? (3) In general, how are you satisfied with this course? The answers were all rated on a 5-point scale. The internal consistency of this scale is 0.96.

#### Qualitative Evaluation of Specific Lessons Based on the Rural P.A.T.H.S. Program

Upon completion of each session, all students were asked to write down their achieved learning outcomes to reflect course effectiveness. The question is “what have you learnt from today’s course?” Students’ answers were coded as “positive” “negative” and “neutral”. An external rater who did not know the research purpose was asked to code 20% of the answers randomly selected from the record. There is only one disagreement among 85 coding units on the positivity of the responses. The interrater reliability was 98.8%. In addition, the answers were also coded into different categories according to the content of the benefits that the participants reflected.

### Evaluation Based on the Service Providers

After the service activities were completed, the implementers were asked to report their perceptions based on the Subjective Outcome Evaluation Scale for service implementers (SOES-SI) (Zhu & Shek, 2021). The scale included three parts. The first part asked service providers about their views on the service activity (10 items) and the performance of their own (10 items), and the answers were rated from 1(strongly disagree) to 6 (strongly disagree). The second part asked service providers to rate their perceived effectiveness of the project on the service recipients from 1(not helpful at all) to 5 (very helpful). The last part is an open question by which the service providers can write down their feedback (such as the gain, the strength, and the difficulty etc.).

## Results

### Evaluation Based on the Service Recipients

The effectiveness of the project was first assessed by analyzing changes in the participants’ PYD qualities and life satisfaction as objective outcome evaluation (see Shek, 2014; Li & Shek, 2019). Only participants who finished the pretest and posttest were included in the subsequent analysis. The demographics of the sample was reported in Table 2. The differences of positive youth development attributes (pretest) between each subgroup were also reported in Table 2. As shown, there is no significant difference between each subgroup except mother education.

**Table 2:** Demographic information of the participants (n = 79)

Variables	Categories	N	%	PYD score (M ± SD)	*P*
Gender	Male	31	39.2%	4.87 ± 0.54	0.36
	Female	48	60.8%	4.98 ± 0.55	
Ethnicity	Han	1	1.3%	4.50	0.40
	Yi	77	98.7%	4.95 ± 0.53	
Grade	5th	18	22.8%	4.99 ± 0.37	0.90
	6th	25	31.6%	4.94 ± 0.65	
	7th	23	29.1%	4.92 ± 0.49	
	8th	11	13.9%	4.84 ± 0.58	
	9th	2	2.5%	5.20 ± 0.35	
Originality	Rural	60	85.7%	4.93 ± 0.51	0.54
	Urban	10	14.3%	5.04 ± 0.55	
Living At School	Yes	43	54.4%	4.88 ± 0.52	0.36
school	No	36	45.6%	4.99 ± 0.53	
Parental marital status	Married	63	79.7%	4.99 ± 0.52	0.33
	Widowed	9	11.4%	4.64 ± 0.57	
	Re-married	3	3.8%	4.87 ± 0.29	
	other	4	5.1%	4.86 ± 0.54	
Father education	Primary school and below	58	78.4%	4.96 ± 0.53	0.20
	Middle school	10	13.5%	4.87 ± 0.37	
	High school/vocational school	4	5.4%	4.66 ± 0.59	
	Junior College	1	1.4%	5.76	
	College and above	1	1.4%	4.16	
Mother Education	Primary school and below	66	85.7%	4.99 ± 0.51	0.01
	Middle school	7	9.1%	4.84 ± 0.43	
	High school/vocational school	4	5.2%	4.13 ± 0.46	
Number of children in the family	2–4	29	36.7%	5.00 ± 0.46	0.18
	5–7	46	58.2%	4.93 ± 0.52	
	8 and above	4	5.1%	4.48 ± 0.91	

To test the changes of participants’ PYD qualities and life satisfaction, we compared participants’ scores of the measures at pretest and posttest using repeated-measure ANOVAs. The results are presented in Table 3. In general, the overall PYD qualities among participants were improved significantly. Specifically, participants’ behavioral competence, clear and positive identity increased significantly after receiving the course. In four higher-order factors, participants showed significant improvement in positive identity, but their changes in other higher-order factors were not significant. The change was significant when we used the total score as the outcome measure. In addition, the demographics of the participants (age, gender and parental education) did not interacted the effectiveness of the program, suggesting that these factors did not have impact on the changes observed. The findings provided support for Hypothesis 1.

**Table 3:** Outcome changes of participants between pretest and posttest

	Pretest	Posttest	
	Mean(*SD*)	Mean(*SD*)	*F*
**PYD (overall level)**	4.94(0.53)	5.02(0.58)	4.075*
**Higher order factors**			
Cognitive-behavioral competences	4.84(0.60)	4.94(0.64)	3.274
Prosocial attributes	5.06(0.58)	5.14(0.75)	1.446
Positive Identity	4.71(0.66)	4.86(0.72)	5.862*
General PYD qualities	4.98(0.56)	5.05(0.57)	2.178
**Primary factors**			
Bonding	5.17(0.57)	5.14(0.64)	0.337
Resilience	5.15(0.77)	5.16(0.68)	0.028
Social competence	4.83(0.73)	4.84(0.75)	0.021
Recognition for positive behavior	4.94(0.69)	4.94(0.73)	0.001
Emotional competence	4.75(0.63)	4.82(0.84)	0.877
Cognitive competence	4.90(0.66)	4.96(0.76)	0.698
Behavioral competence	4.78(0.77)	5.00(0.74)	8.368**
Moral competence	4.67(0.80)	4.82(0.78)	3.375
Self-determination	4.86(0.77)	4.87(0.72)	0.037
Self-efficacy	4.90(0.68)	4.96(0.80)	0.497
Clear and positive identity	4.45(0.77)	4.66(0.81)	6.137*
Beliefs in the future	4.96(0.72)	5.06(0.75)	1.946
Prosocial involvement	5.01(0.64)	5.06(0.78)	0.449
Prosocial norms	5.11(0.62)	5.21(0.80)	1.867
Spirituality	5.46(1.41)	5.68(1.16)	3.639
Life satisfaction	4.22(0.88)	4.24(0.92)	0.058

Participants’ responses to SOES-SR were analyzed to assess their subjective satisfaction with the course content and the course implementers. The mean score of their perception of the course content, teacher and effectiveness of the course is 5.12 (*SD* = 0.67), 5.40 (*SD* = 0.59), and 4.31 (*SD* = 0.64), respectively, indicating the very positive perceptions of the course content, the teachers as well as the course effectiveness. As majority of the responses were positive and the percentages of positive responses were much higher than the negative responses (Table 4), the findings provided support for Hypothesis 2.

**Table 4:** The percentage of students with positive evaluations to the project

Subjective evaluation	Positive responses (N [%])	
**Perception of Course Qualities**		
The objectives of the curriculum are very clear		76(96.2%)
The design of the curriculum is very good.		76(96.2%)
The activities were carefully arranged.		76(96.2%)
The classroom atmosphere was very pleasant.		76(96.2%)
There was much peer interaction amongst the students.		76(96.2%)
I participated actively during lessons.		65(84.4%)
I was encouraged to do my best.		75(94.9%)
The learning experience I encountered enhanced my interest towards the lessons.		76(96.2%)
Overall speaking, I have very positive evaluation of the program.		71(89.9%)
On the whole, I like this curriculum very much.		76(96.2%)
**Perception of Teachers’ Qualities**		
The lecturer(s) had a good mastery of the curriculum.		74(94.9%)
The lecturer(s) was (were) well prepared for the lessons.		77(98.7%)
The teaching skills of the lecturer(s) were good.		75(94.9%)
The lecturer(s) showed good professional attitudes.		78(98.7%)
The lecturer(s) was (were) very involved.		76(96.2%)
The lecturer(s) encouraged students to participate in the activities.		77(97.5%)
The lecturer(s) cared for the students.		79(100%)
The lecturer(s) was (were) ready to offer help to students when needed.		76(96.2%)
The lecturer(s) had much interaction with the students.		76(96.2%)
Overall speaking, I have very positive evaluation of the lecturer(s). the lecturer(s).		77(98.7%)
**Perception of the Effectiveness of the Course**		
It has enhanced my social competence.		74(96.1%)
It has improved my ability in expressing and handling my emotions. my emotions.		78(98.7%)
It has encouraged me to strengthen my collection with family, teachers and other participants.		74(93.7%)
It has increased my competence in making sensible and wise choices. and wise choices.		72(93.5%)
It has helped me to distinguish right from wrong.		76(96.1%)
It has strengthened my resilience in adverse conditions.		74(93.7%)
It has strengthened my self-confidence.		72(93.5%)
It has helped me to face the future with a positive attitude.		73(92.4%)
It has helped me reflect on life.		72(92.3%)
It has enhanced my analytical ability.		75(97.4%)
It has enhanced my ability of refusing bad influence.		76(97.4%)
It has helped me cultivate compassion and care for others. others.		74(96.1%)
It has enhanced my understanding of myself.		75(97.4%)
It has encouraged me to participate in and care for the community.		72(93.5%)
It has promoted my sense of responsibility in serving the society.		76(96.2%)
It has promoted my overall development.		77(97.5%)
**Overall satisfaction**		
On the whole, are you satisfied with this course?		76(96.2%)
Will you suggest your friends to take this course?		72(92.3%)
Will you participate in similar courses again in the future? future?		70(89.7%)

After each lesson, students were asked to write down their views on what they had learned in their own words. Students’ response was coded as “positive” “negative” and “neutral” responses. Results showed that most students provided positive responses to each course (Table 5), which gives support to Hypothesis 3.

**Table 5:** The number of students who provided different responses to the courses

Perceptions of Course Qualities (PC)	Positive Responses (n [%])	Negative Responses (n [%])	Neutral Responses (n [%])	Missing values (n)	Valid values (n)
Course2	81(88.0)	0(0.0)	11(12.0)	3	92
Course3	86(95.6)	0(0.0)	4 (4.4)	5	90
Course4	78(91.8)	0(0.0)	7 (8.2)	10	85
Course5	86(100)	0(0.0)	0 (0.0)	9	86
Course6	81(97.6)	0(0.0)	2 (2.4)	12	83

In addition, the qualitative evaluation was then further categorized into several categories based on the course content (Table 6). The most frequently reported benefits are related to self-efficacy (“learned the way to success”, 93%), active community involvement (“increased awareness of responsibility for community development”, 80.7%) and emotional competence (“found good ways to deal with bad emotions”, 78.3%). For example, in the category of self-efficacy, one student reflected on how to work hard to achieve his/her goals: “Every word of the teacher in the class has inspiration. The teacher said that only hard people deserve a better life, and in the near future through their own efforts may achieve some successful methods, of course, they have to insist.”

**Table 6:** Categorization of perceived benefits of service recipients

Subcategory	Benefits	Number of cases (N[%])
Emotional competence	Enhanced ability in recognizing and handling emotions (e.g. disappointment, anger)	68(73.9%)
	found good ways to deal with bad emotions (e.g. Find a reasonable way to express emotions; Learn to communicate; Share with others; Do no harm to others; drain your emotions)	72(78.3%)
Bonding	Better understanding of parents	40 (44.4%)
	Learned to love and take care of others	28 (31.1%)
	Enhanced collaborative capability	12 (13.3%)
Resilience	Be more proactive in asking for help when facing adversity	48 (53.3%)
	Understood that parents were important protective factors for them	14 (15.6%)
Self-efficacy	Learned the way to success (e.g. Have a goal; be brave; have faith; self-confidence; be optimistic; Work hard; Communicate more with teachers; perseverance; manage yourself)	80 (93.0%)
Beliefs in the future	Provoked thinking for the future	46 (53.5%)
Active community involvement	Increased awareness of responsibility for community development	67 (80.7%)
	Put forward specific measures for the construction of hometown (e.g. protect the environment; develop tourism; protect national culture)	48 (57.8%)
General gains	Felt happy	21 (22.8%)
	Enhance the ability of self-recognition	16 (17.4%)
	Made lots of friends	22 (23.9%)

In addition, we also analyzed whether students’ subjective outcome evaluation was associated with their PYD qualities at posttest. After controlling the demographic information and the pretest PYD qualities, regression analyses showed that participants’ change in posttest PYD quality significantly predicted their subjective perceptions of the subject (*β* = 0.31, *p* < 0.001, *η*^2^ = 0.07) (Table 7). The findings supported Hypothesis 4.

**Table 7:** Regression of post-test PYD on subjective evaluation (n = 79)

Predictors	Model 1			Model 2			Model 3		
	*B(SE)*	*t*	*β*	*B(SE)*	*t*	*β*	*B(SE)*	*t*	*β*
Control variables									
Gender	0.24 (0.15)	1.59	0.21	-0.07 (0.10)	-0.70	-0.06	-0.14 (0.09)	-1.44	-0.12
Originality	-0.1 (0.22)	-0.75	-0.10	-0.31 (0.14)	-2.19*	-0.19	-0.17 (0.13)	-1.30	-0.11
Living at school	0.25 (0.15)	1.67	0.22	0.01 (0.10)	0.10	0.01	0.03 (0.09)	0.34	0.03
Father education	0.05 (0.13)	0.38	0.06	0.09 (0.08)	1.16	0.12	0.04 (0.08)	0.48	0.05
Mother education	-0.38 (0.19)	-2.04*	-0.34	-0.14 (0.12)	-1.10	-0.12	-0.02 (0.12)	-0.15	-0.02
Parental Marriage	-0.10 (0.11)	-0.87	-0.12	0.13 (0.08)	1.71	0.16	0.10 (0.07)	1.43	0.12
Predictors									
PYD(pre)				0.94(0.10)	9.09***	0.84	0.79(0.10)	7.66***	0.71
Subjective evaluation							0.31(0.09)	3.50***	0.31
R^2^_adj_	0.059			0.613			0.678		
F(df1, df2)	1.664(6, 57)			15.260***(1, 56)			17.554***(1, 55)		

*p < 0.05, **p < 0.01, ***p < 0.001

### Evaluation Based on the Service Providers

Subjective outcome evaluation findings based on the service providers are shown in Table 8. All the seven service providers completed the questionnaire and showed 100% positive evaluation of the course quality, their performance and the effectiveness of the course for the participants. The perceived benefits of the course from the service providers showed that the project helped them understand service-learning and positive youth development (Table 9). In addition, they also experienced personal gains from the service, such as the enhancement of expression skills, teaching ability and self-growth. The findings supported Hypothesis 5.

**Table 8:** The percentage of service providers with positive evaluations to the project

Subjective evaluation	Positive responses (N[%])	
**Perception of course qualities**		
The objectives of the curriculum are very clear.		7(100%)
The design of the curriculum is very good.		7(100%)
The activities were carefully arranged.		7(100%)
The classroom atmosphere was very pleasant.		6(85.7%)
There was much peer interaction among the participants.		7(100%)
I participated actively during lessons.		7(100%)
The curriculum has a strong theoretical foundation.		7(100%)
The learning experience I encountered enhanced my interest towards the lessons.		7(100%)
Overall speaking, I have very positive evaluation of the program.		7(100%)
On the whole, I like this curriculum very much.		7(100%)
**Perception of oneself**		
I had a good mastery of the curriculum.		7(100%)
I was well prepared for the lessons.		7(100%)
My teaching skills were good.		7(100%)
I showed good professional attitudes.		7(100%)
I was very involved.		7(100%)
I learnt a lot from teaching.		7(100%)
I cared for the students.		7(100%)
I was ready to offer help to students when needed.		7(100%)
I had much interaction with the participants.		7(100%)
Overall speaking, I have very positive evaluation of myself.		7(100%)
**Perception of the Effectiveness to the participants**		
It has enhanced participants’ social competence.		7(100%)
It has improved participants’ ability in expressing and handling their emotions.		7(100%)
It has encouraged participants to strengthen their collection with family, teachers and other participants.		7(100%)
It has increased participants’ competence in making sensible and wise choices.		7(100%)
It has helped participants to distinguish right from wrong.		7(100%)
It has strengthened participants’ resilience in adverse conditions.		7(100%)
It has strengthened participants’ self-confidence.		7(100%)
It has helped participants to face the future with a positive attitude.		7(100%)
It has helped participants reflect on life.		7(100%)
It has enhanced participants’ analytical ability.		7(100%)
It has enhanced participants’ ability of refusing bad influence.		7(100%)
It has helped participants cultivate compassion and care for others.		7(100%)
It has enhanced participants understanding of theirself.		7(100%)
It has encouraged participants to participate in and care for the community.		7(100%)
It has promoted participants’ sense of responsibility in serving the society.		7(100%)
It has promoted participants’ overall development.		7(100%)
**Overall satisfaction**		
Will you suggest students or clients to take this curriculum?		7(100%)
Will you participate in similar courses again in the future?		7(100%)
On the whole, do you think the implementation of this curriculum will improve your professional growth (e.g. improve your work skills)?		7(100%)

**Table 9:** Categorization of perceived benefits of service providers

Benefits	Students	Total
Improved understanding of service-learning	1,2,6	3
Improved understanding of the concept of positive youths development	1,2,5,7	4
Improved self-expression skills	3,4	2
Enhanced self-growth	1,7	2
Improved teaching ability	5	1

## Discussion

The current study is a pioneer attempt to implement a service-learning project to provide service to Chinese minority adolescents in poverty areas using the rural version of Tin Ka Ping Rural P.A.T.H.S. Program (TKP Rural P.A.T.H.S.). The service-learning subject was found to bring positive changes to the service recipients’ positive development attributes. The finding is consistent with the positive outcomes of previous service-learning subjects regarding service recipients (d’Arlach et al., 2009; Shek, Yang et al., 2021) and provided empirical evidence that service-learning can be well implemented in rural areas to promote positive changes of Yi adolescents.

Triangulation has been recognized as an important way to improve the credibility and validity of research findings (Ammenwerth et al., 2003; Noble & Heale, 2019). As such, the present study used mixed-methods to triangulate the effectiveness of the service-learning project. The evaluations included quantitative and qualitative measures to gauge the perception of the project from both the service recipients and service providers. First, in terms of the service recipients, the present study used objective and subjective evaluations to assess the subject effectiveness. The objective evaluation used the one-group pretest-posttest design to test the change of the service recipients’ positive youth development attributes. Results suggested that the project has improved the Yi adolescents’ overall positive youth development attributes. The finding was consistent with other positive youth development programs provided to adolescents from different backgrounds (Zhu & Shek, 2020a, b). The subjective evaluation included two kinds of analyses: quantitative analysis of the subjective evaluation scale competed by the adolescents and qualitative analysis of the daily feedback of them. The analysis on answer of the subjective scale shows that more than 90% of the Yi adolescents believed that the course was very effective. Besides the quantitative evaluation, the qualitative responses to each session given by the Yi adolescents were also mostly positive, the adolescents reported their improvements in emotional competence, bonding, and self-efficacy etc. In addition, the recipients’ change in of their overall positive youth development attributes at posttest was correlated to subjective outcome evaluation. In short, the positive effect of the project on the Yi adolescents were proved by both the objective and subjective evaluation in terms of the perception of the service recipients.

Second, from the perspective of the service implementers, the service was perceived as very positive in terms of course content, service providers’ self-perception and effectiveness. The service providers also gained a better understanding of service-learning and the concept of positive youth development. This finding provides support for our Hypothesis 5 and was consistent with other research on service-learning in mainland China (see Peng et al., 2009). In conclusion, the above findings lend support to our five hypotheses and further indicate that this service-learning subject implemented in rural China is successful in terms of the positive changes of the recipients and the positive evaluation from the service providers.

The present study used various evaluations to access the rural version of Tin Ka Ping P.A.T.H.S. Program’s impact on service-learning recipients. As expected, the Yi adolescents who attended the 7-day rural version of Tin Ka Ping P.A.T.H.S. Program (TKP-PATHS-R) showed significant improvement in their overall positive youth development scores. The finding is also consistent with previous Tin Ka Ping P.A.T.H.S. Program (Shek et al., 2014; Zhu & Shek, 2020a, b), which suggested that the revised rural version of the Tin Ka Ping P.A.T.H.S. program seems to fit well in the rural context. In the future, we should further test the utility of the rural version of Tin Ka Ping P.A.T.H.S. program in other rural areas in China.

In view of the positive findings of the service recipients, there is the question of why such positive changes take place. There are several explanations. First, with the absence of a control group, the findings may be due to natural maturation. However, the subjective and qualitative findings suggest that the participants actually felt benefits of the program. Second, the participants may simply “please” the implementers. While this is a possibility for the subjective outcome and qualitative evaluation data, “pleasing” the implementers are extremely difficult for pretest-posttest changes. Third, the findings can be interpreted as showing the positive program effects. It is noteworthy that all evaluation findings in the study point to the same direction and researchers have commonly interpret related findings in terms of positive program effects.

It is noteworthy that there are several limitations of the present study. First, the sample was not large in this study although power analysis showed that the sample size was sufficient for statistical tests of significance. It would be illuminating to recruit more adolescents to the project in the future. Second, due to practical difficulties of implementing the project in minority communities, the project time is limited. It would be more insightful to provide the project over a longer period so that we might got a better understanding of the long-term impact of the service-learning program. Third, we only measured the pre- and post-changes of Yi adolescents’ positive youth attributes, it would be illuminating to collect the evaluation data over time and include more objective indicators such as the academic grades and teacher’s assessment. Besides, as recruiting control participants in this Yi community was extremely difficult, we adopted a pre-experimental design to test the objective changes of the Yi adolescents. Though the findings of the objective evaluation was consistent with previous studies of positive youth development programs (Shek & Law, 2014; Zhu & Shek, 2020a, b) it would be helpful to add a control group in future study if possible.
